# Diagnosing Zika virus infection against a background of other flaviviruses: Studies in high resolution serological analysis

**DOI:** 10.1038/s41598-019-40224-2

**Published:** 2019-03-06

**Authors:** Sören Hansen, Sven-Kevin Hotop, Oumar Faye, Oumar Ndiaye, Susanne Böhlken-Fascher, Rodrigo Pessôa, Frank Hufert, Christiane Stahl-Hennig, Ronald Frank, Claus-Peter Czerny, Jonas Schmidt-Chanasit, Sabri S. Sanabani, Amadou A. Sall, Matthias Niedrig, Mark Brönstrup, Hans-Joachim Fritz, Ahmed Abd El Wahed

**Affiliations:** 1 Division of Microbiology and Animal Hygiene, University of Goettingen, Goettingen, 37075 Germany; 2 Department of Chemical Biology, Helmholtz-Zentrum für Infektionsforschung, Inhoffenstrasse 7, 38124 Braunschweig, Germany; 3 Deutsches Zentrum für Infektionsforschung (DZIF), Standort Hannover-, Braunschweig, Germany; 4 Institut Pasteur de Dakar, Dakar, Senegal; 5 Laboratory of Dermatology and Immunodeficiencies, LIM-56, Department of Dermatology, Tropical Medicine Institute of São Paulo, University of São Paulo, São Paulo, Brazil; 6 Institute of Microbiology and Virology, Brandenburg Medical School Fontane, Senftenberg, Germany; 7 Deutsches Primatenzentrum GmbH, Leibniz-Institut für Primatenforschung, Unit of Infection Models, Göttingen, Germany; 8 AIMS Scientific Products GmbH, Berlin, Germany; 9 Bernhard Nocht Institute for Tropical Medicine, WHO Collaborating Centre for Arbovirus and Hemorrhagic Fever Reference and Research, Hamburg, Germany; 10 German Centre for Infection Research (DZIF), partner site Hamburg-Luebeck-Borstel, Hamburg, Germany; 11 Robert Koch Institut, Nordufer 20, 13353 Berlin, Germany; 12 Akademie der Wissenschaften zu Göttingen, Theaterstraße 7, 37073 Göttingen, Germany

## Abstract

Zika virus (ZIKV) is a mosquito-borne flavivirus. Homologous proteins of different flaviviruses display high degrees of sequence identity, especially within subgroups. This leads to extensive immunological cross-reactivity and corresponding problems for developing a ZIKV-specific serological assay. In this study, peptide microarrays were employed to identify individual ZIKV antibody targets with promise in differential diagnosis. A total of 1643 overlapping oligopeptides were synthesized and printed onto glass slides. Together, they encompass the full amino acid sequences of ZIKV proteomes of African, Brazilian, USA, and French Polynesian origins. The resulting ZIKV scanning microarray chips were used to screen three pools of sera from recent Zika outbreaks in Senegal and Cape Verde, in Brazil, and from overseas travelers returning to the EU. Together with a mixed pool of well characterized, archived sera of patients suffering from infections by dengue, yellow fever, tick-borne encephalitis, and West Nile viruses, a total of 42 sera went into the study. Sixty-eight antibody target regions were identified. Most of which were hitherto unknown. Alignments and sequence comparisons revealed 13 of which could be classified as *bona fide* ZIKV-specific. These identified antibody target regions constitute a founding set of analytical tools for serological discrimination of ZIKV from other flaviviruses.

## Introduction

Zika virus (ZIKV), a mosquito-borne flavivirus, was first isolated from sentinel Rhesus macaques in the Zika forest of Uganda in 1947^1^. The first human case was recorded in Nigeria in 1954. Before 2007, many small-scale epidemics were recorded in Africa and Asia^2^. Yap island (Micronesia) was the first area outside Africa and Asia to confirm human ZIKV infection^3^. Around one-tenth of the population of French Polynesia encountered the infection during the 2013 outbreak^4^. In 2015, the Brazilian ministry of health estimated around 440,000 to 1,300,000 individuals in Brazil that may have contracted the infection. The potential of ZIKV spreading to other countries is high with almost one-fourth of the human population worldwide being at risk of encountering ZIKV infection. In regions like Europe, ZIKV transmission risk is present through high mobility and global connectivity^5^.

Clinical signs of ZIKV infection are ranging from asymptomatic to mild headaches, fever, skin rashes and joint pain, which usually last a few days. The symptoms are unspecific and are frequently misdiagnosed as caused by dengue or chikungunya virus^6^. ZIKV is transmitted mainly by *Aedes* mosquitoes. Many reports discuss two additional ZIKV infection pathways: sexual intercourse and from a pregnant woman to her fetus^7,8^. ZIKV RNA was detected in the amniotic fluid of a pregnant woman^9^ and in the brain of an aborted fetus afflicted with microcephaly^10^. Moreover, ZIKV was linked to increased numbers of Guillian-Barré Syndrome cases in affected countries^11^.

ZIKV RNA can be detected in blood and saliva during the first five days of onset of symptoms, up to 21 days in urine and for up to one year in semen^12–15^. Longer persistence of anti-ZIKV antibodies in serum (IgM: up to two months; IgG: two years)^16,17^ extends the practicable diagnostic time window. On the other hand, high degrees of sequence identity of homologous proteins and concomitant immunological cross-reactivity among all flaviviruses, in particular within the various subgroups, have been reported^18,19^. These facts profoundly complicate serological diagnosis, especially in areas, where ZIKV and dengue virus are co-circulating and/or yellow fever vaccine is applied routinely^20,21^. Still, serological differentiation of ZIKV from other flaviviruses remains desirable and some progress towards resolving the difficulties has been announced^22–24^. This present study introduces a new approach resting on the identification, in larger numbers, of ZIKV-specific antibody targets. Specifically, we report comparative deconvolution of complex B cell responses against ZIKV and other flavivirus by screening corresponding sera with a microarray chip made up of overlapping peptides covering the entire amino acid sequence of the ZIKV genomic polyprotein. As a result, 13 short linear stretches of polypeptide sequence, scattered throughout the viral proteome, were identified that exhibit exclusive reactivity towards ZIKV antisera.

## Results

A (series of) positive array spot(s) indicates an antibody target and positions it within a protein sequence. In the context of the overlapping peptides method, the term ‘antibody target’ denotes a stretch of polypeptide chain of an antigen encompassing either a single linear B cell epitope or a locally clustered set thereof. In the latter case, it is called an antibody target region (ATR)^25^. Since a spots series as such often does not allow distinguishing between single epitope and ATR, the latter term is occasionally also used as a synonym of the generic name “antibody target” with epitopes regarded as extreme cases within that class.

Compiling a comprehensive list of antibody targets (epitopes, ATRs) participating in B cell responses to ZIKV infection and singling out from it the ZIKV-specific subset will, so the working hypothesis, enable diagnosis of ZIKV infection against a background of extensively cross-reacting other flaviviruses (*e.g*. dengue, yellow fever, tick-borne encephalitis, and West Nile virus). As shown earlier^25^, the necessary deconvolution of complex B cell responses to the level of contributions made by individual short stretches of polypeptide chain can be achieved experimentally by peptide microarray techniques. Comparing ATRs resulting from inspection of ZIKV and non-ZIKV flaviviral antisera will reduce the former to the ZIKV-specific subset. From this, discriminating immunochemical reagents can be derived.

### Immunochemical experiments

**Figure 1:**
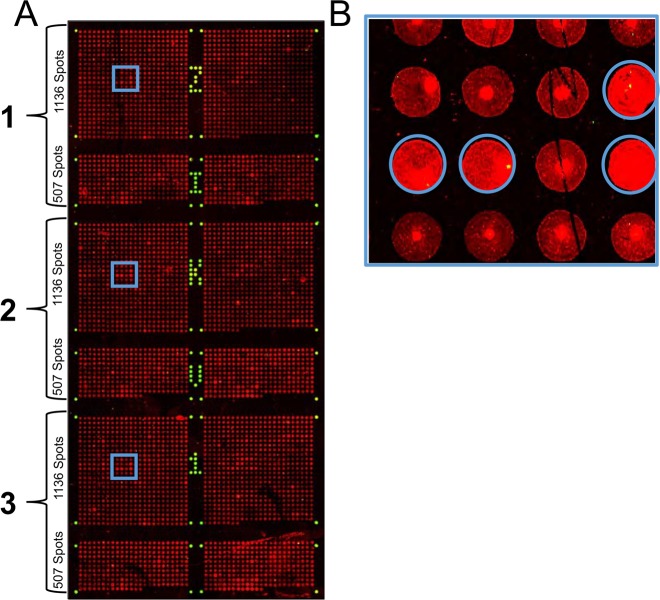
The ZIKV peptide microarray chip. (Panel A) Layout: Each chip hosts three identical arrays, each of which encompasses 1643 pentadekapeptides: 1136 covering the complete ZIKV Africa (AAV34151) polyprotein with an overlap of consecutive individual peptides of 12 amino acid residues plus 507 pentadekapeptides accommodating divergent sequences present in other ZIKV isolates (Brazil, USA, Senegal, French Polynesia). In addition, there are 96 biotin spots (green), which serve as internal positive controls and as markers of array boundaries (ref.^23^). (Panel B) Identification of positive signals: The criterion for a positive response is bright red fluorescence spread evenly across the entire spot area (examples marked by a blue circle).

**Figure 2:**
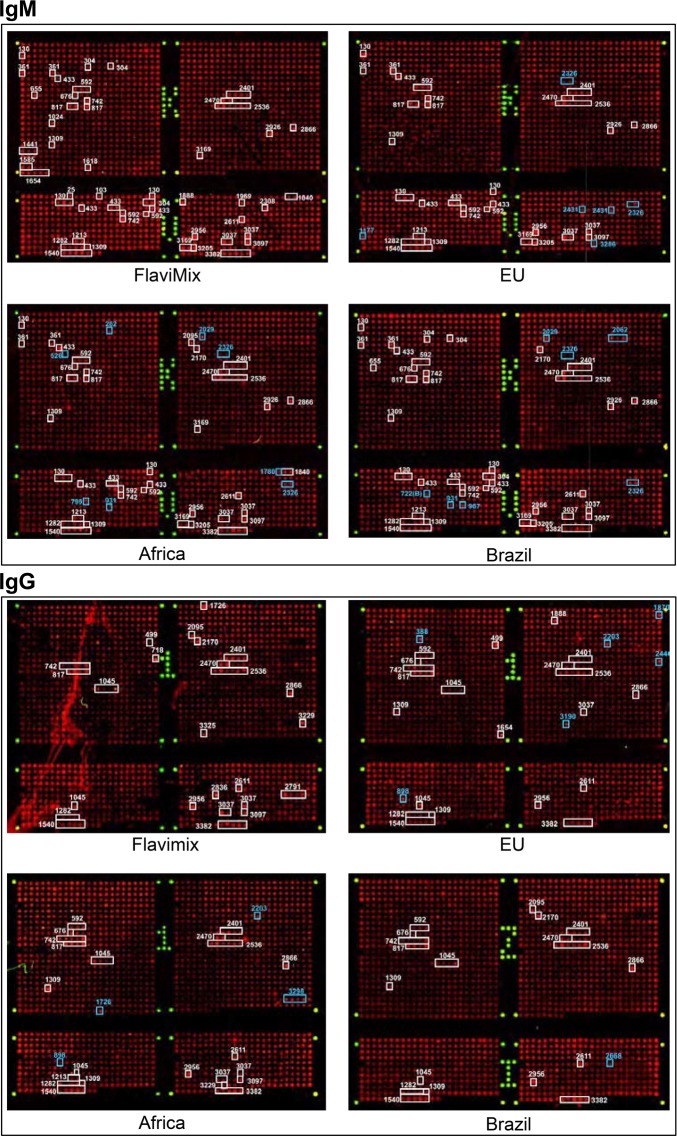
Original screening data (examples). Array spots showing positive reaction are boxed in. White boxes: Responses seen with FlaviMix pool (either exclusive or in combination with one or several ZIKV serum pools). Blue boxes: Responses seen only with ZIKV serum pools (“ZIKV-exclusives”). ATR numbers are stated next to corresponding boxes (Fig. 3). For experimental details refer to Materials and Methods.

Three pools of sera from ZIKV-infected individuals and one mixed pool of anti-flavivirus sera were prepared and screened for serological footprints (separate experiments for IgM and IgG) employing peptide microarray chips (Fig. 1). Two sets of original data are displayed in Fig. 2. Positively responding array spots (Fig. 1) delineate antibody targets (white and blue frames in Fig. 2).

### ATRs identified: Overview and crude classification

**Figure 3:**
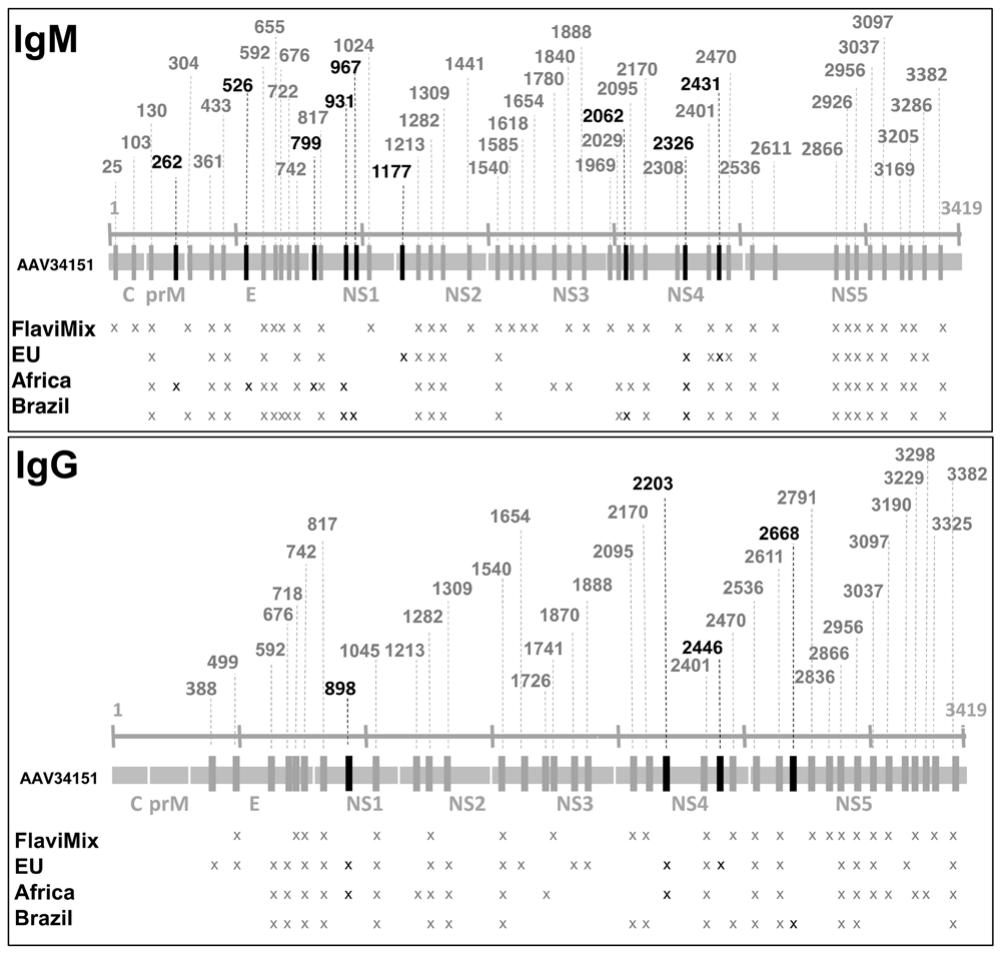
Mapping ATRs to ZIKV polyprotein. The horizontal bar represents the 3419 residue long unprocessed translation product of the ZIKV genome (Genbank accession number AAV34151). Marks along the grey line are spaced in intervals of 500 amino acid residues. ATRs are named by the number of their residue lying most closely to the N-terminus (Supplementary Table S1). ATRs marked black (class I ATRs, Fig. 5 and Supplementary Table S2) exclusively reacted with one or more ZIKV pools. ATRs marked grey reacted with FlaviMix pool and with zero to three ZIKV pools.

**Figure 4:**
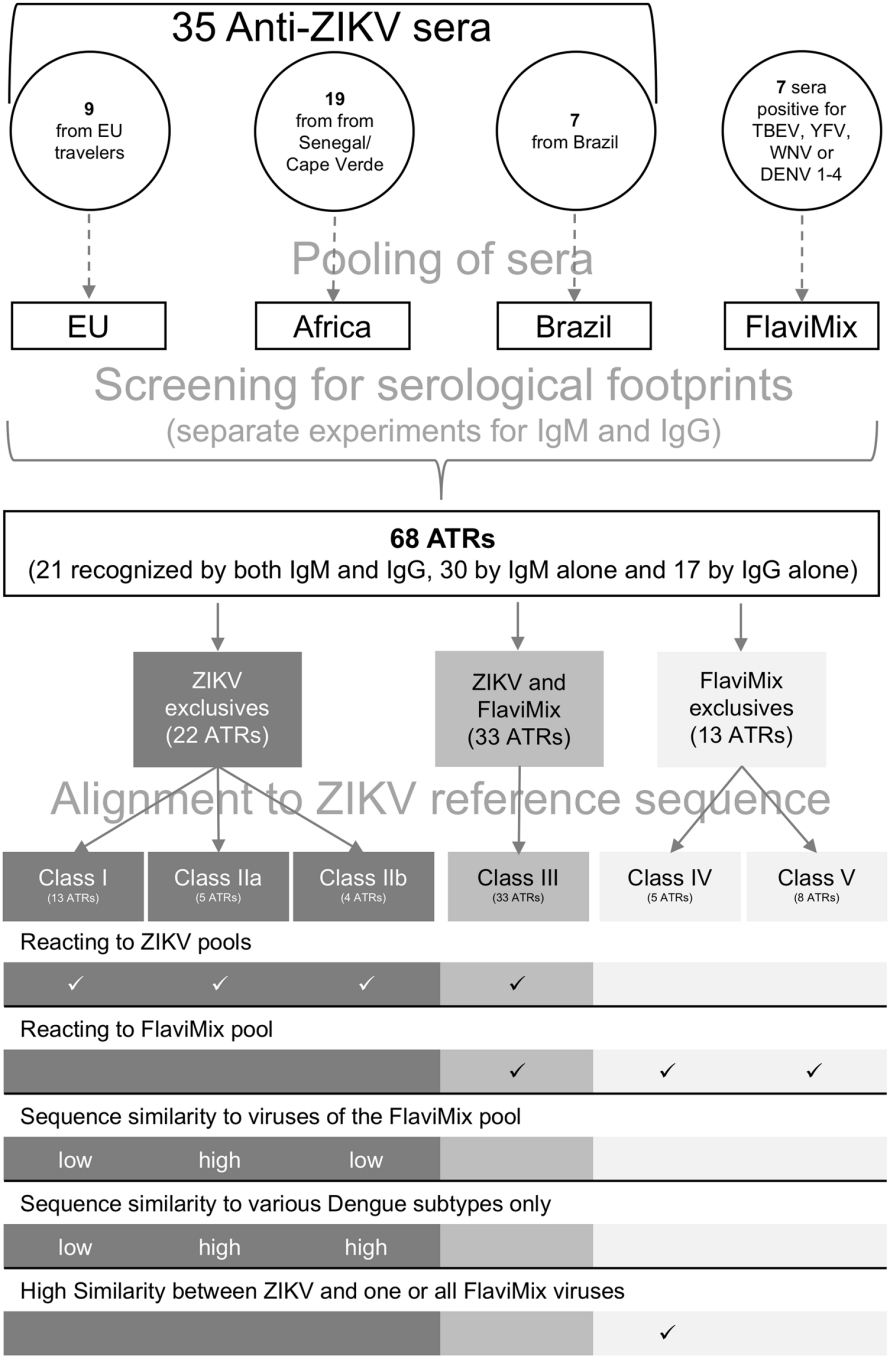
Workflow of the study.

**Figure 5:**
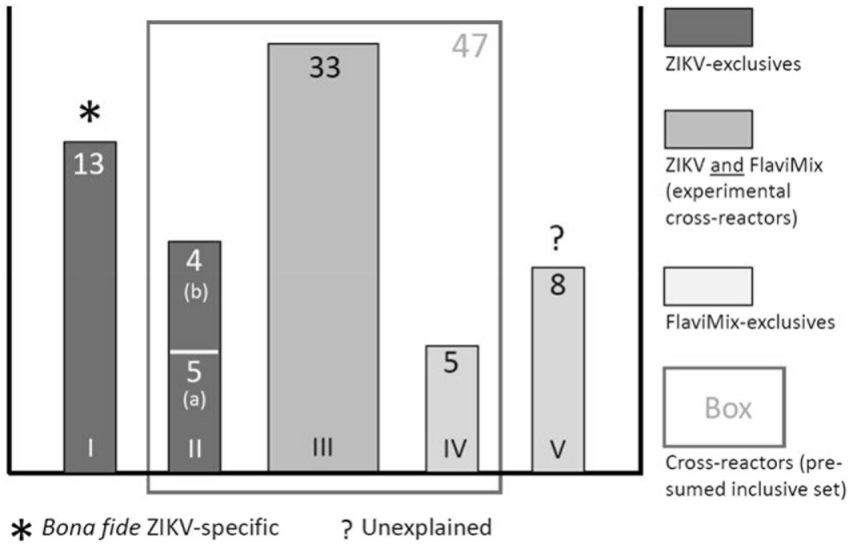
Classification of 68 experimentally identified ATRs. Roman numerals: Name of class. Arabic numerals: Number of ATRs in respective class. Class I: ZIKV-exclusives with low similarity to homologous sequences of FlaviMix viruses (residue identity <80%). Class II: Same with high similarity (residue identity ≥80%). IIa: Similarity present in sequences of all viruses making up FlaviMix pool. IIb: Same but similarity only observed for Dengue subtypes. Class III: ATRs eliciting antibodies responding to both ZIKV and FlaviMix sera pools. Class IV: FlaviMix-exclusives with high similarity between ZIKV and FlaviMix. Class V: FlaviMix-exclusives with no clear-cut location on polyprotein map of any virus represented in FlaviMix. Numbers of cases are graphically represented as bar areas. For usage of the term “exclusive” refer to main text.

The experiments uncovered 68 ATRs, most of which were hitherto unknown. Of these, 21 were recognized by both IgM and IgG, 30 by IgM alone and 17 by IgG alone (Figs 2–4 and Supplementary Table S1). The targets are scattered across the entire proteome with no striking preference of envelope over non-envelope proteins (Fig. 3). From antibodies directed against the latter, no contribution to virus neutralization would be expected, but in the context of diagnosis they are equally useful. Twenty-two ATRs reacted exclusively with ZIKV sera (Figs 4 and 5, classes I and II), 13 exclusively with FlaviMix (classes IV and V) and 33 with both FlaviMix and at least one ZIKV pool (class III). Sequences of corresponding stretches of polypeptide chain are compiled in Supplementary Tables S2 and S3. Alignments and sequence comparisons prompted sub-division of the ZIKV-exclusive and the FlaviMix-exclusive sets as explicated in detail below. Note that with “exclusive” we merely describe an observation in the present assay – in contrast to “specific” as an intrinsic property of a given antibody target.

Strings of amino acid residues displaying exclusive immunochemical reactivity towards ZIKV sera would be expected to differ substantially between ZIKV and the respective homologous sites of viruses making up the FlaviMix pool. This was true for 13 of 22 cases (class I, Supplementary Table S2, Fig. 5). Class I cases (such as ATR #2062, listed in Table 1) are characterized by a low to intermediate level of sequence identity between ZIKV and other flaviviruses across the respective antigen (NS4 in the case of ATR #2062) and a similarly low or lower degree of identity within the actual target region under consideration. The 13 class I ATRs are distributed among all ZIKV antigens – with the exception of antigens C and NS3 (Fig. 3). For the remaining nine cases (class II), sequence identity across the entire length of the respective antigen is low to intermediate but within the target region it is high between either ZIKV and all viruses of the FlaviMix pool (class IIa, five cases, represented in Table 1 by ATR #388) or ZIKV and the various Dengue subtypes only (class IIb, four cases, represented in Table 1 by ATR #2029).

**Table 1:** Properties of ZIKV-exclusive ATRs.

ATR and Class	Virus (accession number)	Id (%) Polyprotein (within Subgroup)	Sequence	Id (%) ATR	Id (%) Antig.	Id (%) Antig. within Subgroup
**ATR #2062** (NS4) [Class I: 13 members] IgM (e,a,B) IgG (e,a,b)	Zika (AAV34151, Africa)	100	DGTTNNTIMEDSVP-AEVWTKY	100	100	
	Zika (AOS90225, USA)	96.4	..............-.....RH	100	96.5	
	Zika (ALU33341, Brazil)	96.4	..............-.....RH	90.9	97.1	
	Zika (AHZ13508, Fr. Polynesia)	96.5	..............-.....RH	90.9	96.6	
	Zika (AHL43504, Senegal)	99.0	..............-.......	90.9	99.1	
	Dengue 1 (NP_059433)	55.5 (98.6)	..ER..QVL.ENMD-V.I...E	40.9	45.8	98.9
	Dengue 2 (NP_056776)	55.5 (97.7)	..VK..Q.L.EN.E-V.I...E	50.0	49.3	98.1
	Dengue 3 (YP_001621843)	56.2 (98.7)	..QR..Q.L.ENMD-V.I...E	45.5	47.0	98.1
	Dengue 4 (NP_073286)	56.3 (98.3)	T.ER..Q.L.ENME-V.I..RE	36.4	47.3	98.8
	Yellow Fever (NP_041726)	46.6 (96.4)	E.PEEHE.LN..GETVKCRAPG	22.7	34.4	96.3
	West-Nile (YP_001527877)	57.3 (98.6)	..PRT...L..NNE-V..I..L	50.0	43.0	98.2
	TBE (NP_043135)	41.5 (95.8)	E.PEA.AVD.A.GDLVTFRSPN	22.7	29.5	96.1
**ATR #388** (E) [Class IIa: 5 members] IgM (e,a,b) IgG (E,a,b)	Zika (AAV34151, Africa)	100	DRGWGNGCGLFGKGS	100	100	
	Zika (AOS90225, USA)	96.4	...............	100	96.4	
	Zika (ALU33341, Brazil)	96.4	...............	100	96.2	
	Zika (AHZ13508, Fr. Polynesia)	96.5	...............	100	94.4	
	Zika (AHL43504, Senegal)	99.0	...............	100	98.9	
	Dengue 1 (NP_059433)	55.5 (98.6)	...............	100	58.9	98.5
	Dengue 2 (NP_056776)	55.5 (97.7)	..............G	93.3	54.5	98.0
	Dengue 3 (YP_001621843)	56.2 (98.7)	...............	100	58.7	98.5
	Dengue 4 (NP_073286)	56.3 (98.3)	..............G	93.3	56.7	98.3
	Yellow Fever (NP_041726)	46.6 (96.4)	...............	100	43.3	96.9
	West-Nile (YP_001527877)	57.3 (98.6)	...............	100	54.0	98.9
	TBE (NP_043135)	41.5 (95.8)	......H........	93.3	39.1	98.8
**ATR #2029** (NS4) [Class IIb: 4 members] IgM (e,A,B) IgG (e,a,b)	Zika (AAV34151, Africa)	100	TFVELMKRGDLPVWL	100	100	
	Zika (AOS90225, USA)	96.4	...............	100	96.5	
	Zika (ALU33341, Brazil)	96.4	...............	100	97.1	
	Zika (AHZ13508, Fr. Polynesia)	96.5	...............	100	96.6	
	Zika (AHL43504, Senegal)	99.0	...............	100	99.1	
	Dengue 1 (NP_059433)	55.5 (98.6)	......R........	93.3	45.8	98.9
	Dengue 2 (NP_056776)	55.5 (97.7)	...D..R........	86.7	49.3	98.1
	Dengue 3 (YP_001621843)	56.2 (98.7)	......R........	93.3	47.0	98.1
	Dengue 4 (NP_073286)	56.3 (98.3)	......R........	93.3	47.3	98.8
	Yellow Fever (NP_041726)	46.6 (96.4)	V.R..VRNC......	60.0	34.4	96.3
	West-Nile (YP_001527877)	57.3 (98.6)	N.L..LRTA......	60.0	43.0	98.2
	TBE (NP_043135)	41.5 (95.8)	H.RH.LTHC.FTP..	33.3	29.5	96.1

Three ATRs exclusively responding to ZIKV sera are illustrated, one representative case of each class I, IIa and IIb (for classification refer to Fig. 5; a comprehensive compilation of all ZIKV-exclusive ATRs and their sequence characteristics is given in Supplementary Table S2).

Id values, if not stated otherwise, denote % sequence identity with reference strain Zika (AAV34151, Africa) of genomic polyprotein, respective antigen and ATR as indicated in column headers. In addition, Id values indicating bandwidths of sequence variation within subgroups of non-ZIKV flaviviruses (polyprotein or individual antigen) are listed. These were derived as follows.

*(i)* A consensus sequence was computed for each subgroup. *(ii)* For each member of the respective subgroup percent residue identity with the consensus sequence was calculated. *(iii)* The mean value of these was defined as “Id within subgroup”. Alignments were performed as described in Materials and Methods. Symbols for provenances of ZIKV-sera are as follows. E: EU pool, A: African pool, B: Brazilian pool. Capital letters (E, A, B) indicate positive signal observed with respective sera pool, while small letters (e, a, b) represent lack of reactivity with either IgM or IgG.

There are thirteen ATRs reacting exclusively with the FlaviMix pool. Five of these (class IV: #718, #1024, #1618, #2836, #3325, Supplementary Table S3) show strong similarity or identity with the respective homologous sites in ZIKV. Eight cases of FlaviMix-exclusive responses remain unexplained (class V: ATRs #25, #103, #1441, #1585, #1741, #1969, #2308, #2791, Supplementary Table S1). Anyhow, class V ATRs lack relevance to the present problem as they will not make any contribution to the development of a practical serological test.

## Discussion

This work marks the entry into a systematic and comprehensive search for antibody targets (epitopes, ATRs) of ZIKV in relation to those of other flaviviruses with the aim of identifying diagnostically useful antigenic sites eliciting ZIKV-*specific* antibodies. Despite the limited number of sera available for our analyses, some remarkable reactivity patterns emerged.

### Antibody targets reacting with ZIKV sera pools exclusively

Among the 68 ATRs, 22 (summarized in Supplementary Table S2) reacted exclusively with one or more ZIKV sera pools, 13 recognized by IgM and 9 by IgG with no overlap of the two sets. This lack of overlap is surprising in view of the fact that the combined processes of affinity maturation and class-switching do not qualitatively change the antigen recognition properties of an antibody: In a notional, infinitely large collection of ZIKV-positive sera, all antibody targets should be represented in both the IgM and the IgG set.

High local sequence conservation of an antigenic site across various virus species immediately suggests cross-reactivity. As cross-reactivity was missing in some cases, an explanation could, in principle, be offered by different antigenicity of the same stretch of polypeptide chain in homologous antigens due to different local three-dimensional structure (high for ZIKV, low for other flaviviruses). Some differences of 3D-structure have indeed been observed among different flaviviruses^26^.

Taken together, the findings corroborate the validity of the rationale underlying this study and the thirteen class I ATRs are the *first bona* fide candidates for being truly ZIKV-specific as required for serologically diagnosing ZIKV infection against a dense background of cross-reactivity with other flaviviruses.

### Cross-reacting antibody targets

Expectations that close serological scrutiny would reveal extensive cross-reactivity between ZIKV and other flaviviruses were borne out: At least 33 of the 68 ATRs identified in ZIKV and FlaviMix cross-react. As argued below, the real number is probably as high as 47 (Fig. 5). All ZIKV antigens carry a multitude of ATRs eliciting cross-reacting antibodies (Fig. 3) – a serious handicap for any serological ZIKV assay resting on whole antigens. Nevertheless, at least one attempt based on whole NS1 antigens of ZIKV and DENV (three cross-reacting ATRs by our count) has been made employing a combination of tests and quantitative signal comparison^23^. Prekumar *et al*. alleviate the cross-reactivity problem by focussing on two isolated domains of the ZIKV envelope protein that combine pronounced antigenicity with low sequence similarity between ZIKV and DENV^22^. Lastly, a competition ELISA based on a ZIKV-specific monoclonal anti-NS1 antibody has shown remarkable selectivity and specificity^21^. The assay, however, is bound to miss detecting ZIKV-infections of individuals that happened not to produce antibodies against that one particular epitope.

### Experimental results compared with epitope prediction

Two studies have been published that aim at predicting B cell epitopes of ZIKV envelope proteins on the basis of theoretical considerations and homology to other flaviviruses^27,28^. Our experiments confirm 18 of these predictions and uncovered 37 additional targets (classes I to III, Fig. 5), not only in non-envelope proteins. On the other hand, 28 predictions could not be verified experimentally. This, however, does not necessarily indicate failed predictions but could alternatively be due, as in cases mentioned above, to a still incomplete collection of experimental targets. For a comprehensive comparison of prediction and experiment refer to Supplementary Table S4.

### Non-saturating sampling of antibody targets

The notion of incomplete experimental representation of ZIKV ATRs in a limited number of human sera turned up in several different contexts. These are as follows. ***(i)*** Nine ATRs (class II) responded only to ZIKV sera despite their occurrence in homologous FlaviMix antigens with identical or very similar sequences. ***(ii)*** Reciprocally: Five ATRs of high sequence similarity between ZIKV and other flaviviruses (class IV) reacted exclusively with the FlaviMix pool. ***(iii)*** Among the 22 ZIKV-exclusive responses there is no overlap between ATRs recognized by the IgM and the IgG set of antibodies. ***(iv)*** Only a subset of epitopes, previously predicted on theoretical grounds^27,28^, could be verified (so far) experimentally.

For every individual one of these four independent observations, incomplete ATR sampling offers an only tentative explanation; the notion, however, gains cogency by giving all of them a common, unified underpinning. As a corollary, two predictions follow: (***a***) Very likely, class II and class IV ATRs are true cross-reactors (Fig. 5). In contrast, there is no reason *a priori* to doubt the ZIKV specificity of the 13 members of class I. (***b***) A fair number of ZIKV ATRs are still to be discovered. This may turn out to be of practical importance in the eventual compilation of a larger set of ZIKV-specific ATRs.

## Conclusion and Outlook

Specific serological identification of ZIKV against a background of cross-reacting other flaviviruses requires use of ZIKV-specific antibody targets. Since, in principle, any single target is represented in an individual B cell response with a probability of less than one, high sensitivity standards can only be met by the combined application of several targets in parallel – each characterized by highest possible antigenicity. In that sense, substantial headway towards the development of a ZIKV-specific diagnostic assay has been made with the discovery of 13 *bona fide* ZIKV-specific ATRs as reported here.

The remaining agenda *en route* to a practical diagnostic procedure such as ELISA, line assay, or bead flow-through assay is as follows. ***(i)*** Still other potentially cross-reacting flaviviruses (*e.g*. Japanese encephalitis and spondweni viruses) need to be included. ***(ii)*** The assignment of the 13 ATRs in class I as ZIKV-specific needs to be further consolidated. ***(iii)*** Additional specific ATRs should be found and ranked with respect to their antigenicity (*i.e*. signal frequency in a set of test sera).

All of the above points can be addressed in a straightforward manner, larger numbers of sera being the only prerequisite lying outside the scope of the present study. The approach builds upon antigen amino acid sequence as the only indispensable knowledge and it is not limited to addressing the cross-reactivity problem of flaviviruses only.

Beyond diagnosis, peptide microarray-based, high resolution serology may contribute to gaining a better understanding of a number of infection-connected problems. Antibody-mediated virus enhancement, for example, is a well-documented phenomenon associated with flaviviruses and epidemiological studies linked Zika outbreaks to increased incidence of Guillain-Barré syndrome, a neurological autoimmune condition known to interact with humoral immunity^29^.

## Materials and Methods

### Serum samples and ethics statement

Samples of 42 well-characterized sera were provided by reference laboratories from Germany (Bernhard Nocht Institute, Hamburg), Senegal (Institut Pasteur de Dakar) and Brazil (University of São Paulo). The FlaviMix sera pool consists of 7 sera directed against dengue 1–4, yellow fever, tick-borne encephalitis and West Nile viruses. The EU pool comprises 9 anti-ZIKV sera from EU travellers returning from Brazil. The Africa pool encompasses 19 anti-ZIKV sera from patients living in Senegal and Cape Verde. The Brazilian pool contains 7 anti-ZIKV sera from patients diagnosed at the University of São Paulo. All sera were either reference sera from the WHO collaboration center (Institut Pasteur de Dakar) or collected from patients during routine medical examinations or surveillance. All samples were collected between 5 to 28 days post onset of fever from patients tested positive with real-time PCR to the respective virus during the acute phase (>5 days). Samples which reacted positive to more than one virus were excluded from the study. Patients had given consent according to national and international ethical regulations. All samples were handled anonymously.

### ZIKV peptide microarray scanning chips

The ZIKV proteome of the African strain is made up of seven antigens (prM not counted as a separate module) with a cumulative length of 3419 amino acid residues^30^. Antigen sequences were broken up into 1136 pentdecapeptides with a consecutive overlap of 12 residues representing the ZIKV African strain (GenBank accession number AAV34151). These were synthesized *via* SPOT synthesis^31^, passed through the SC2 process^32^ and spotted onto glass microscope slides (AIMS Scientific Products GmbH, Berlin, Germany) as illustrated in Fig. 1A (upper part of each of three identical arrays displayed in Fig. 1A). Each of the (smaller) lower array parts accommodates 507 additional pentadecapeptides that cover sequence stretches differing from the reference sequence in other ZIKV strains (Brazil, ALU33341; USA, AOS90225; Senegal, AHL43504; French Polynesia, AHZ13508). In addition, 96 spots of a biotin/(β-alanine)_2_/cellulose conjugate were deposited on the chips as positive controls and for marking array boundaries. After staining, they light up green (Fig. 1A).

### Chip screening procedure

In each individual experiment, a ZIKV microarray chip was washed with absolute ethanol for 3 min, then three times for 3 min each with TBS (50 mM Tris base, 150 mM NaCl, HCl ad pH 7.6). After incubation with blocking buffer (TBS, 2% casein, 0.1% Tween-20) overnight, the chip was washed with T-TBS (TBS, 0.1% Tween-20) for 3 min. Thereafter, 70 µl of any of the four serum pools, diluted 120-fold, were added onto the chip. The chip was incubated in a humidified chamber at 4 °C overnight. Thereafter, the chip was washed three times for 5 min each with T-TBS. To visualize the binding of primary antibodies, the following was added onto the chip: 60 µl of blocking buffer containing Cy3-conjugated mouse anti-biotin and Alexa Fluor 647-conjugated goat antibody directed against either human IgG or human IgM (240-fold dilution of stock solutions prepared according to instructions of the commercial supplier, Jackson Immunoresearch Laboratories, West Grove, PA, USA). The chip was kept in a humidified chamber at room temperature for 1.5 hours. Subsequently, the chip was washed twice for 5 min with T-TBS, three times with distilled water for 5 min each, and dried by dipping into acetonitrile for 10 sec before visualization with an Agilent DNA microarray scanner. Each serum pool was tested in triplicate (three individual chips, corresponding to nine arrays total). For unknown reasons, all four sera pools produced atypically high levels of background which precluded application of the previously developed, automated spot calling procedure^25^. Therefore, array patterns were visually inspected and positive responses were identified in two steps – with the burden of safeguarding against false positives laid predominantly on stringent selection in step 1: *(i)* Individual signals were called positive if they showed bright red fluorescence spread uniformly across the entire spot area (Fig. 1B). *(ii)* A spot was taken as indicating an antibody target, if it passed step 1 in at least two arrays of the same chip with at least two out of three separate chips.

### Sequence alignment

Bioinformatics: Experimentally determined ATRs were aligned to viral polyproteins using the pairwise alignment tool of the GENEIOUS program package (Biomatters Ltd, Auckland, New Zealand). Query sequences were composed of the respective ATR plus fifty amino acid residues each on both sides – all taken from the reference ZIKV strain (GenBank accession number: AAV34151).

### Ethical statement

No ethical approval was required as all sera were either reference sera from the WHO collaboration center (Institut Pasteur de Dakar) or collected from patients during routine medical examinations or surveillance. All samples were handled anonymously.

## Supplementary information

Supplementary Dataset

## Data Availability

All data produced during this study were included in the manuscript. Original data files are available upon request to the corresponding author.
