# Supplementary material for: Diagnosing Zika virus infection against a background of other flaviviruses: Studies in high resolution serological analysis
Source: Sci Rep. 2019 Mar 6;9:3648. doi: 10.1038/s41598-019-40224-2 (PMC6403343; doi:10.1038/s41598-019-40224-2)
Supplement: Supplementary file 1 — Supplementary Dataset [file 41598_2019_40224_MOESM1_ESM.pdf]

Supplementary Material

Titel: Diagnosing Zika virus infection against a background of other flaviviruses: Studies in high resolution serological analysis

Sören Hansen[1#], Sven-Kevin Hotop[2&3#], Oumar Faye[4], Oumar Ndiaye[4], Susanne Böhlken-Fascher[1], Rodrigo Pessôa[5], Frank Hufert[6], Christiane Stahl-Hennig[7], Ronald Frank[8], Claus-Peter Czerny[1], Jonas Schmidt-Chanasit[9&10], Sabri S. Sanabani[5], Amadou A. Sall[4], Matthias Niedrig[11], Mark Brönstrup[2&3], Hans-Joachim Fritz[12¶], Ahmed Abd El Wahed[1¶*]

1) Division of Microbiology and Animal Hygiene, University of Goettingen, 37075, Germany

2) Department of Chemical Biology, Helmholtz-Zentrum für Infektionsforschung, Inhoffenstrasse 7, 38124 Braunschweig, Germany

3) Deutsches Zentrum für Infektionsforschung (DZIF), Standort Hannover-Braunschweig, Germany

4) Institut Pasteur de Dakar, Dakar, Senegal

5) Laboratory of Dermatology and Immunodeficiencies, LIM-56, Department of Dermatology, Tropical Medicine Institute of São Paulo, University of São Paulo, São Paulo, Brazil

6) Institute of Microbiology and Virology, Brandenburg Medical School Fontane, Senftenberg, Germany

7) Deutsches Primatenzentrum GmbH, Leibniz-Institut für Primatenforschung, Unit of Infection Models, Göttingen, Germany

8) AIMS Scientific Products GmbH, Berlin, Germany

9) Bernhard Nocht Institute for Tropical Medicine, WHO Collaborating Centre for Arbovirus and Hemorrhagic Fever Reference and Research, Hamburg, Germany

10) German Centre for Infection Research (DZIF), partner site Hamburg-Luebeck-Borstel, Hamburg, Germany

11) Robert Koch Institut, Nordufer 20, 13353 Berlin, Germany

12) Akademie der Wissenschaften zu Göttingen, Theaterstraße 7, 37073 Göttingen, Germany

#Equal contribution

¶Joint senior authors

*Correspondence to abdelwahed@gwdg.de

Table S1: Experimentally identified ATRs: Comprehensive List.

ATR Nr.	Spot number	Sequence Number	Accession Code	Sequence	IgG FlaviMix Detection	IgG Eu Detection	IgG Africa Detection	IgG Brazil Detection	IgM FlaviMix Detection	IgM Eu Detection	IgM Africa Detection	IgM Brazil Detection
25	1145	25-39	2,3,4	SPFGGLKRLPAGLLL					x			
103	1150	103-117	2,3,4	EKRRGADTSVGIVG					x			
130	1159	130-172	2,3,4	RRGSAYYMYLDRNDA					x			
	49		1,5	ISFATTLGVNKCHVQ						x		
	1166		2,3	PTTLGMNKCYIQIMD					x	x		x
	1168		2,3,4	NKCYIQIMDLGHMCD					x	x	x	x
262	88	262-276	1,5	VAIAWLLGSSTSQKV							x	
304	1182	304-348	3	GMSGGTWVDIVLEHG					x			
	1183		3	GGTWVDIVLEHGGCV					x			
	108		1,2,3,4,5	TVMAQDKPTVDIELV					x			x
	112		1,2,3,4,5	ELVTTTVSNMAEVRS								x
361	121	361-390	1,2,3,4,5	DSRCPTQGEAYLDKQ					x			x
	126		1,2,3,4,5	SDTQYVCKRTLVDRG					x	x	x	x
388	130	388	1,2,3,4,5	DRGWGNGCGLFGKGS		x						
433	1200	433-469	5	VHGSQHSGMIVNDIG						x	x	
	1201		5	SQHSGMIVNDIGHET						x		
	1195		2	TGHETDENRAKVEIT							x	x
	151		1	AKVEVTPNSPRAEAT						x		
	1207		5	RAKVEVTPNSPRAEA					x			
499	167	499-513	1,2,3,4,5	VHKEWFHDIPLPWHA	x	x						
526	176	526-540	1,2,3,4,5	EALVEFKDAHAKRQT							x	
592	1225	592-627	5	SLCTAAFTFTKVPAE							x	
	202		1,2,3,4,5	PAETLHGTVTVEVQY		x	x	x	x	x	x	x
	203		1,2,3,4,5	TLHGTVTVEVQYAGT		x	x	x	x	x	x	x
	204		1,2,3,4,5	GTVTVEVQYAGTDGP		x	x	x	x	x	x	x
	1229		2,3	TVEVQYAGTDGPCKV							x	x
655	219	655-669	1,2,3,4,5	TENSKMMLELDPPFG					x			
676	226	676-690	1,5	GVGDKKITHHWHRSG		x	x	x	x		x	x
718	240	718	1,5	GSVGGVFNSLGKGIH	x							
722	1244	722-735	2,3,4	GGALNSLGKGIHQIF								x
742	248	742-771	1,2,3,4,5	LFGGMSWFSQILIGT			x					
	252		1,5	IGTLLVWLGLNTKNG	x	x	x	x	x	x	x	x
	1250		2,3,4	LLMWLGLNTKNGSIS							x	
799	1268	799-813	2,3,4	SKKETRCGTGVFVYN							x	
817	273	817-840	1,2,3,4,5	AWRDRYKYHPDSPRR	x	x	x	x	x	x	x	x
	274		1,2,3,4,5	DRYKYHPDSPRRLAA	x	x	x	x	x	x	x	x
	276		1,2,3,4,5	PDSPRRLAAAVKQAW	x	x	x	x	x	x	x	x
898	1287	898	5	VNGLPHGWKAWGKSY		x	x					
931	1296	931-945	2,3,4,5	KECPLKHRAWNSFLV							x	x
967	1298	967-981	2,3,4	LECDPAVIGTAVKGK								x
1024	342	1024-1038	1,5	DGVEESDLIIPKSLA					x			
1045	349	1045-1077	1,5	NTREGYRTQVKGPWH	x	x	x	x				
	353		1,2,3,4,5	PWHSEELEIRFEECP	x	x	x	x				
	1314		2,3,4,5	LEIRFEECPGTKVHV		x						
1177	1329	1177-1191	2,3	STSMAVLVAMILGGF						x		
1213	1338	1213-1230	2,3,4	GGDVAHLALIAAFKV			x		x	x	x	x
	1339		2,3,4	VAHLALIAAFKVRPA			x		x		x	x
1282	1360	1282-1305	5	RAMAVPRTDNIALAI	x	x	x	x	x	x	x	x
	1362		5	RTDNIALAILAALTP	x	x	x	x	x	x	x	x
	1363		5	NIALAILAALTPLAR	x	x	x	x	x	x	x	x
1309	1364	1309-1326	2,3,4,5	LVAWRAGLATCGGFM		x	x	x	x	x	x	x
	438		1	WRAGLATCGGIMLLS			x		x		x	
1441	481	1441-1461	1,2,3,4,5	RLDVALDESGDFSLV					x			
	482		1,5	VALDESGDFSLVEED					x			
	483		1,5	DESGDFSLVEEDGPP					x			
1540	1384	1540-1566	2,3,4	QEGVFHTMWHVTKGS	x	x	x	x			x	x
	1385		2,3,4	VFHTMWHVTKGSALR	x	x	x	x			x	x
	1386		2,3,4	TMWHVTKGSALRSGE	x	x	x	x			x	x
	1387		2,3,4	HVTKGSALRSGEGRL	x	x	x	x	x	x	x	x
	1388		2,3,4	KGSALRSGEGRLDPY	x	x	x	x	x	x	x	x
1585	529	1585-1602	1,5	AAWDGLSEVQLLAVP					x			
	530		1,5	DGLSEVQLLAVPPGE					x			
1618	540	1618-1632	1,2,3,4,5	DGDIGAVALDYPAGT					x			
1654	552	1654-1683	1,2	IKNGSYVSAITQGKR		x						
	553		1,5	GSYVSAITQGKREEE					x			
	554		1,5	VSAITQGKREEETPV					x			
	555		1,5	ITQGKREEETPVECF					x			
	556		1,5	GKREEETPVECFEPS					x			
	557		1,2,3,4,5	EEETPVECFEPSMLK					x			
1726	567	1726-1740	1,2,3,4,5	VAAEMEEALRGLPVR			x					
1741	581	1741-1755	1,2,3,4,5	YMTTAVNVTHSGTEI	x							
1780	1408	1780-1794	2,3,4	YIMDEAHFTDPSSIA							x	
1840	1409	1840-1857	3	ERAWSSGFDWVTDYS							x	
	1410		3	WSSGFDWVTDYSGKT						x		
1870	624	1870-1884	1,2,3,4,5	IAACLTKAGKRVIQL		x						

1888	630	1888-1902	1,5	TFETEFQKTKNQEWD		x						
	1415		2,3,4	TFETEFQKTKHQEWD					x			
1969	1425	1969-1983	2,3,4	YLYGGGCAETDEDHA					x			
2029	677	2029-2043	1,2,3,4,5	TFVELMKRGDLPVWL							x	x
2062	688	2062-2082	1,2,3,4,5	DGTTNNTIMEDSVPA								x
	690		1,5	TIMEDSVPAEVWTKY								x
2095	699	2095-2109	1,2,3,4,5	ARVCSDHAALKSFKE	x			x	x		x	
2170	724	2170-2184	1,2,3,4,5	IMLLGLLGTVSLGIF	x			x	x		x	x
2203	735	2203-2217	1,2,3,4,5	LGASAWLMWLSEIEP		x	x					
2308	1453	2308-2322	2,3,4	YAALTTFITPAVQHA					x			
**2326**	776	2326-2358	1,2,3,4,5	SYNNYSLMAMATQAG							x	
	777		1,2,3,4,5	NYSLMAMATQAGVLF							x	
	1457		2,3,4,5	VLFGMGKGMPFYAWD						x		
	1458		2,3,4,5	GMGKGMPFYAWDFGV							x	x
2401	801	2401-2424	1,2,3,4,5	QKRTAAGIMKNPVVD	x	x	x	x	x	x	x	x
	802		1,2,3,4,5	TAAGIMKNPVVDGIV	x	x	x	x	x	x	x	x
	803		1,2,3,4,5	GIMKNPVVDGIVVTD	x	x	x	x	x	x	x	x
	804		1,2,3,4,5	KNPVVDGIVVTDIDT	x	x	x	x	x			x
2431	1473	2431-2445	5	VEKKMGQVLLIAVAV						x		
2446	816	2446-2460	1,5	SSAVLLRTAWGWGEA		x						
	1478			SSAILSRTAWGWGEA					x			
2470	824	2470-2487	1,2,3,4,5	TLWEGSPNKYWNSST	x			x	x	x	x	x
	825		1,2,3,4,5	EGSPNKYWNSSTATS	x				x	x	x	x
2536	847	2536-2568	1,2,3,4,5	SALEFYSYKKSGITE					x	x	x	x
	848		1,2,3,4,5	SYKKSGITEVCREEA	x	x	x	x	x	x	x	x
	849		1,2,3,4,5	KSGITEVCREEARRA	x	x	x	x		x		
	850		1,2,3,4,5	ITEVCREEARRALKD	x	x	x	x				
	851		1,2,3,4,5	VCREEARRALKDGVA	x	x	x	x			x	x
	852		1,2,3,4,5	EEARRALKDGVATGG	x	x	x	x			x	x
2611	1497	2611-2625	2,3,4	RKVQEVKGYTKGGPG	x	x	x	x	x		x	x
2668	1502	2668-2682	2,3,4	SSPEVEEARTLRVLS				x				
2791	1529	2791-2817	2,3,4	MKIIGNRIERIRSEH	x							
	1533		2,3,4	SEHAETWFFDENHPY	x							
2836	1540	2836-2850	2,3,4,5	SLINGVVRLLSKPWD	x							
2866	956	2866-2880	1,2,3,4,5	YGQQRVFKEKVDTRV	x	x	x	x	x	x	x	x
2926	976	2926-2940	1,2,3,4,5	LGAIFEEEKEWKTAV					x	x	x	x
2956	1561	2956-2970	2,3,4	REHHLRGECQSCVYN	x	x	x	x	x	x	x	x
3037	1570	3037-3069	2,3,4	EEMSRIPGGRMYADD	x				x	x		x
	1590		5	EEMSQAPGGKMYADD	x		x		x	x	x	x
	1591		5	SQAPGGKMYADDTAG							x	
	1019		1,5	WDTRISKFDLENEAL		x						
3097	1594	3097-3111	2,3,4	VLRPAEKGKTVMDII	x		x		x	x	x	x
3169	1607	3169-3192	5	GWDGLKRMAVSGDDC								
	1608		5	GLKRMAVSGDDCVVK					x	x	x	x
	1060		1,2,3,4,5	VSGDDCVVKPIDDRF					x	x	x	x
3190	1064	3190-3204	1,2,3,4,5	DRFAHALRFLNDMGK		x						
3205	1609	3205-3219	2,3,4	VRKDTQEWKPSTGWD					x	x	x	x
3229	1616	3229-3246	2,3,4	HHFNKLHLKDGRSIV			x					
	1078		1,5	NKLYLKDGRSIVVPC	x							
3286	1619	3286-3300	2,3,4	HRRDLRLMANAICSS						x		
3298	1100	3298-3321	1,5	CSAVPVDWVPTGRTT			x					
				VPVDWVPTGRTTWSI			x					
				DWVPTGRTTWSIHGK			x					
				PTGRTTWSIHGKGEW			x					
3325	1109	3325	1,5	EDMLMVWNRVWIEEN	x							
3382	1638	3382-3405	2,3	KNTVNMVRRIIGDEE			x		x			
	1639		4	KNTVNMVRRIIGEEE	x		x		x			
	1640		3	VNMVRRIIGEEEKYM	x	x	x	x	x		x	x
	1641		3	VRRIIGEEEKYMDYL	x	x	x	x	x		x	x
	1642		3	IIGEEEKYMDYLSTQ	x	x	x	x	x		x	x

Acession Number	Origin	Accession Code
AAV34151	Africa	1
AHZ13508	French Polyn.	2
ALU33341	Brasil	3
AOS90225	USA	4
AHL43504	Africa	5

ATR	ZIKA-exclusive
Sequence Number	reacted with IgM
Sequence Number	reacted with IgG
Sequence Number	reacted with IgM and IgG

Table S2: ZIKV-exclusive ATRs (classes I, IIa, IIb). Entries of class I ATRs are marked by grey highlighting of header cells. For other explanations see legend to table 1.

Virus (accession number)	Id (%) Polyprotein (within Subgroup)	Class I: ATR #262 (preM)					Class IIa: ATR #388 (E)					Class I: ATR #526 (E)					Class IIb: ATR #722 (E)				
		IgM (e,A,b)	IgG (e a,b)	Id (%) ATR	Id (%) Antig.	Id (%) within Subgroup	IgM (e,a,b)	IgG (E,a,b)	Id (%) ATR	Id (%) Antig.	Id (%) within Subgroup	IgM (e,A,b)	IgG (e,a,b)	Id (%) ATR	Id (%) Antig	Id (%) within Subgroup	IgM (e,a,B)	IgG (e,a,b)	Id (%) ATR	Id (%) Antig.	Id (%) within Subgroup
Zika (AAV34151, Africa)	100	VAIAWLLGSSTSQKV		100	100		DRGWGNGCGLFGKGS		100	100		EALVEFKDAHAKRQT		100	100		GGVFNSLGKGIHQIF		100	100	
Zika (AOS90225, USA)	96.4	A..............		93.3	94.5		....		100	96.4		....		100	96.4		..AL...........		86.7	96.4	
Zika (ALU33341, Brazil)	96.4	A..............		93.3	94.5		....		100	96.2		....		100	96.2		..AL...........		86.7	96.2	
Zika (AHZ13508, Fr. Polynesia)	96.5	A..............		93.3	94.5		....		100	94.4		....		100	94.4		..AL...........		86.7	94.4	
Zika (AHL43504, Senegal)	99.0	....		100	100		....		100	98.9		....		100	98.9		....		100	98.9	
Dengue 1 (NP_059433)	55.5 (98.6)	LFL.HAI.T.IT..G		33.3	43.5	98.7	....		100	58.9	98.5	DL..T..T....K.E		60.0	58.9	98.5	....T.V..L.....		80.0	58.9	98.5
Dengue 2 (NP_056776)	55.5 (97.7)	AIL.YTI.TTHF.RA		20.0	39.9	97.4	..............G		93.3	54.5	98.0	.T..T..NP...K.D		60.0	54.5	98.0	....T.I..AL..V.		66.7	54.5	98.0
Dengue 3 (YP_001621843)	56.2 (98.7)	LFL.HYI.T.LT...		40.0	42.3	99.0	....		100	58.7	98.5	.L..T..N....K.E		66.7	58.7	98.5	...L.....MV....		80.0	58.7	98.5
Dengue 4 (NP_073286)	56.3 (98.3)	GFM.YMI.QTGI.RT		20.0	44.6	99.3	..............G		93.3	56.7	98.3	.RM.T..VP.....D		60.0	56.7	98.3	..L.T....AV..V.		66.7	56.7	98.3
Yellow Fever (NP_041726)	46.6 (96.4)	LT..Y.V..NMT.R.		46.7	37.3	97.6	....		100	43.3	96.9	HH....EPP..ATIR		40.0	43.3	96.9	..F.T.V.....TV.		66.7	43.3	96.9
West-Nile (YP_001527877)	57.3 (98.6)	AV.G.M...N.M.R.		53.3	42.9	99.0	....		100	54.0	98.9	.T.M..EEP..TK.S		46.7	54.0	98.9	....T.V..AV..V.		66.7	54.0	98.9
TBE (NP_043135)	41.5 (95.8)	.TVV..TLE.VVTR.		33.3	35.5	95.0	......H........		93.3	39.1	98.8	.R....GAP..VKMD		46.7	39.1	98.8	..FLS.I..AV.TVL		40.0	39.1	98.8

Virus (accession number)	Id (%) Polyprotein (within Subgroup)	Class I: 799 (NS1)					Class I: ATR #898 (NS1)					Class I:ATR # 931 (NS1)					Class I: ATR #967 (NS1)				
		IgM (e,A,b)	IgG (e,a,b)	Id (%) ATR	Id (%) Antig.	Id (%) within Subgroup	IgM (e,a,b)	IgG (E,A,b)	Id (%) ATR	Id (%) Antig.	Id (%) within Subgroup	IgM (e,A,B)	IgG (e,a,b)	Id (%) ATR	Id (%) Antig.	Id (%) within Subgroup	IgM (e,a,B)	IgG (e,a,b)	Id (%) ATR	Id (%) Antig.	Id (%) within Subgroup
Zika (AAV34151, Africa)	100	SKKETRCGTGVFIYN		100	100		VNELPHGWKAWGKSY		100	100		KECPLEHRAWNSFLV		100	100		LECDPAVIGTAVKGR		100	100	
Zika (AOS90225, USA)	96.4	............V..		93.3	97.2		....		100	97.2		.....K.........		93.3	97.2		..............K		93.3	97.2	
Zika (ALU33341, Brazil)	96.4	............V..		93.3	97.2		..............H		93.3	97.2		.....K.........		93.3	97.2		..............K		93.3	97.2	
Zika (AHZ13508, Fr. Polynesia)	96.5	............V..		93.3	97.5		....		100	97.5		.....K.........		93.3	97.5		..............K		93.3	97.5	
Zika (AHL43504, Senegal)	99.0	..R............		93.3	99.7		..G............		93.3	99.7		.....K.........		93.3	99.7		....		100	99.7	
Dengue 1 (NP_059433)	55.5 (98.6)	KGR.LK..S.I.VT.		40.0	54.0	98.4	PM.HKYS..S...AK		40.0	54.0	98.4	P...DNQ....IWE.		53.3	54.0	98.4	QV..HRLMSA.I.DS		26.7	54.0	98.4
Dengue 2 (NP_056776)	55.5 (97.7)	KN..LK..S.I..TD		46.7	54.8	97.6	PT..KYS..T...AK		46.7	54.8	97.6	A...NTN.....LE.		60.0	54.8	97.6	VF..SKLMSA.I.DN		26.7	54.8	97.6
Dengue 3 (YP_001621843)	56.2 (98.7)	KG..LK..S.I.VT.		46.7	55.7	98.7	PM..KYS..T...AK		46.7	55.7	98.7	P...SAS....VWE.		53.3	55.7	98.7	QL..HRLMSA...DE		33.3	55.7	98.7
Dengue 4 (NP_073286)	56.3 (98.3)	.G..LK..S.I.VVD		46.7	53.7	97.7	.SD.KYS..T...AK		46.7	53.7	97.7	S...N.R.....LE.		60.0	53.7	97.7	EV..HRLMSA.I.DQ		26.7	53.7	97.7
Yellow Fever (NP_041726)	46.6 (96.4)	G.R.LK..D.I..FR		46.7	47.6	96.7	RDG.QY...T...NL		46.7	47.6	96.7	....FSN.V....QI		60.0	47.6	96.7	ID..GSIL.A..N.K		40.0	47.6	96.7
West-Nile (YP_001527877)	57.3 (98.6)	.RQ.L...S....H.		60.0	56.3	98.2	TEK.EI........I		60.0	56.3	98.2	....TQN.....LE.		66.7	56.3	98.2	T...SKI......NN		60.0	56.3	98.2
TBE (NP_043135)	41.5 (95.8)	ERM.L...E.LV.WR		40.0	43.5	98.5	GKDIRVS..S..H.M		33.3	43.5	98.5	S.....R.KTGV.T.		53.3	43.5	98.5	H...TG.M.A...NG		53.3	43.5	98.5

Virus (accession number)	Id (%) Polyprotein (within Subgroup)	Class I: ATR #1177 (NS2)					Class IIa: ATR #1726 (NS3)					Class IIa: ATR # 1780 (NS3)					Class IIb: ATR #1870 (NS3)				
		IgM (E,a,b)	IgG (e,a,b)	Id (%) ATR	Id (%) Antig.	Id (%) within Subgroup	IgM (e,a,b)	IgG (e,A,b)	Id (%) ATR	Id (%) Antig.	Id (%) within Subgroup	IgM (e,A,b)	IgG (e,a,b)	Id (%) ATR	Id (%) Antig.	Id (%) within Subgroup	IgM (e,a,b)	IgG (E,a,b)	Id (%) ATR	Id (%) Antig.	Id (%) within Subgroup
Zika (AAV34151, Africa)	100	STSMAVLVVMILGGF		100	100		VAAEMEEALRGLPVR		100	100		NIMDEAHFTDPSSIA		100	100		IAACLTKAGKRVIQL		100	100	
Zika (AOS90225, USA)	96.4	........A......		93.3	97.0		....		100	98.0		Y..............		93.3	98.0		....		100	98.0	
Zika (ALU33341, Brazil)	96.4	........A......		93.3	97.0		....		100	97.8		Y..............		93.3	97.8		....		100	97.8	
Zika (AHZ13508, Fr. Polynesia)	96.5	........A......		93.3	97.0		....		100	98.0		Y..............		93.3	98.0		....		100	98.0	
Zika (AHL43504, Senegal)	99.0	....		100	99.2		....		100	100		....		100	100		....		100	100	
Dengue 1 (NP_059433)	55.5 (98.6)	TGTL..FLLLTM.QL		20.0	29.8	97.6	..S..A...K.M.I.		66.7	65.8	99.1	I..........A...		86.7	65.8	99.1	..N..R.N....V..		73.3	65.8	99.1
Dengue 2 (NP_056776)	55.5 (97.7)	LLVAVSF.TL.T.NM		20.0	32.3	97.0	.............I.		93.3	66.4	98.3	I..........A...		86.7	66.4	98.3	.....R.N..K....		80.0	66.4	98.3
Dengue 3 (YP_001621843)	56.2 (98.7)	AGVFFTF.LLLS.QI		13.3	30.6	98.2	.........K...I.		86.7	62.2	99.1	I..........A...		86.7	62.2	99.1	..N..R.N..K....		73.3	62.2	99.1
Dengue 4 (NP_073286)	56.3 (98.3)	LVVVIT.CAI....L		33.3	31.8	97.5	.............I.		93.3	67.0	98.7	IV...........V.		80.0	67.0	98.7	..N..R.S..K....		73.3	67.0	98.7
Yellow Fever (NP_041726)	46.6 (96.4)	VGGVVL.GA.LV.QV		20.0	32.0	98.2	.LS..K..FH..D.K		53.3	51.8	97.9	I.......L..A...		80.0	51.8	97.9	M..S.R....S.VV.		60.0	51.8	97.9
West-Nile (YP_001527877)	57.3 (98.6)	PAILIA.L.LVF..I		26.7	41.0	98.1	.....A.......I.		86.7	67.7	98.7	FV.........A...		80.0	67.7	98.7	..L..QR...K.V..		66.7	67.7	98.7
TBE (NP_043135)	41.5 (95.8)	WGGIV..ALLVT.MV		20.0	21.2	92.5	.LK...R..N.KR..		60.0	45.6	97.0	A......W...H...		80.0	45.6	97.0	..RT.RQK..S..C.		53.3	45.6	97.0

Virus (accession number)	Id (%) Polyprotein (within Subgroup)	Class IIb: ATR #2029 (NS4)					Class I: ATR #2062 (NS4)					Class I: ATR #2203 (NS4)				
		IgM (e,A,B)	IgG (e,a,b)	Id (%) ATR	Id (%) Antig.	Id (%) within Subgroup	IgM (e,a,B)	IgG (e,a,b)	Id (%) ATR	Id (%) Antig.	Id (%) within Subgroup	IgM (e,a,b)	IgG (E,A,b)	Id (%) ATR	Id (%) Antig.	Id (%) within Subgroup
Zika (AAV34151, Africa)	100	TFVELMKRGDLPVWL		100	100		DGTTNNTIMEDSVP-AEVWTKY		100	100		LGASAWLMWLSEIEP		100	100	
Zika (AOS90225, USA)	96.4	....		100	96.5		..............-.....RH		100	96.5		....		100	96.5	
Zika (ALU33341, Brazil)	96.4	....		100	97.1		..............-.....RH		90.9	97.1		....		100	97.1	
Zika (AHZ13508, Fr. Polynesia)	96.5	....		100	96.6		..............-.....RH		90.9	96.6		....		100	96.6	
Zika (AHL43504, Senegal)	99.0	....		100	99.1		..............-.......		90.9	99.1		....		100	99.1	
Dengue 1 (NP_059433)	55.5 (98.6)	......R........		93.3	45.8	98.9	..ER..QVL.ENMD-V.I...E		40.9	45.8	98.9	VI..SA.L.MASV..		40.0	45.8	98.9
Dengue 2 (NP_056776)	55.5 (97.7)	...D..R........		86.7	49.3	98.1	..VK..Q.L.EN.E-V.I...E		50.0	49.3	98.1	IITASI.L.YAQ.Q.		26.7	49.3	98.1
Dengue 3 (YP_001621843)	56.2 (98.7)	......R........		93.3	47.0	98.1	..QR..Q.L.ENMD-V.I...E		45.5	47.0	98.1	VI..SGML.MA.VPL		26.7	47.0	98.1
Dengue 4 (NP_073286)	56.3 (98.3)	......R........		93.3	47.3	98.8	T.ER..Q.L.ENME-V.I..RE		36.4	47.3	98.8	IAVASG.L.VA..Q.		33.3	47.3	98.8
Yellow Fever (NP_041726)	46.6 (96.4)	V.R..VRNC......		60.0	34.4	96.3	E.PEEHE.LN..GETVKCRAPG		22.7	34.4	96.3	MAGCGY..F.GGVK.		26.7	34.4	96.3
West-Nile (YP_001527877)	57.3 (98.6)	N.L..LRTA......		60.0	43.0	98.2	..PRT...L..NNE-V..I..L		50.0	43.0	98.2	..VATFFC.MA.VPG		26.7	43.0	98.2
TBE (NP_043135)	41.5 (95.8)	H.RH.LTHC.FTP..		33.3	29.5	96.1	E.PEA.AVD.A.GDLVTFRSPN		22.7	29.5	96.1	.L..LL.L.AGGVGY		33.3	29.5	96.1

Virus (accession number)	Id (%) Polyprotein (within Subgroup)	Class I: ATR #2326 (NS4)					Class I: ATR #2431 (NS4)					Class I: ATR #2446 (NS4)				
		IgM (E,A,B)	IgG (e,a,b)	Id (%) ATR	Id (%) Antig.	Id (%) within Subgroup	IgM (E,a,b)	IgG (e,a,b)	Id (%) ATR	Id (%) Antig.	Id (%) within Subgroup	IgM (e,a,b)	IgG (E,a,b)	Id (%) ATR	Id (%) Antig.	Id (%) within Subgroup
Zika (AAV34151, Africa)	100	SYNNYSLMAMATQAGVLFGMGKGMPFMHGDLGV		100	100		VEKKMGQVLLIAVAI		100	100		SSAVLLRTAWGWGEA		100	100	
Zika (AOS90225, USA)	96.4	..........................YAW.F..		87.9	96.5		V		93.3	96.5		...I.S.........		86.7	96.5	
Zika (ALU33341, Brazil)	96.4	..........................YAW.F..		87.9	97.1		....		100	97.1		...I.S.........		86.7	97.1	
Zika (AHZ13508, Fr. Polynesia)	96.5	..........................YAW.F..		87.9	96.6		V		93.3	96.6		...I.S.........		86.7	96.6	
Zika (AHL43504, Senegal)	99.0	..........................YAW.F..		87.9	99.1		....		100	99.1		....		100	99.1	
Dengue 1 (NP_059433)	55.5 (98.6)	TTA.I..T.I.N..AI.M.LD..W.ISKM.I..		45.5	45.8	98.9	F..QL..IM.LILCT		33.3	45.8	98.9	.QIL.M..T.ALC.S		40.0	45.8	98.9
Dengue 2 (NP_056776)	55.5 (97.7)	.SV.V..T.I.N..T..M.L...W.LSKM.I..		54.5	49.3	98.1	F..QL...M.LVLCV		33.3	49.3	98.1	TQVLMM..T.ALC..		33.3	49.3	98.1
Dengue 3 (YP_001621843)	56.2 (98.7)	.TA.V..A.I.N..V..M.LD..W.ISKM....		54.5	47.0	98.1	F..QL...M.LVLCA		33.3	47.0	98.1	VQLL.M..S.ALC..		40.0	47.0	98.1
Dengue 4 (NP_073286)	56.3 (98.3)	TSA.L..A.I.N..A..M.L...W.LHRM....		54.5	47.3	98.8	F..QL...M.LVLCA		33.3	47.3	98.8	GQLL.M..T.AFC.V		33.3	47.3	98.8
Yellow Fever (NP_041726)	46.6 (96.4)	E.G.L..SGI.QS.S..SF.D..I...KMNIS.		45.5	34.4	96.3	Y...LALY..L.LSL		40.0	34.4	96.3	A.VAMC..PFSLA.G		26.7	34.4	96.3
West-Nile (YP_001527877)	57.3 (98.6)	D.I.T..TSINV..SA..TLAR.F..VDVGVSA		33.3	43.0	98.2	MQ..V..IM..L.SL		46.7	43.0	98.2	AAV.VNPSVKTVR..		20.0	43.0	98.2
TBE (NP_043135)	41.5 (95.8)	KIQQLVNS.V.SG.QAMRDL.G.A..FGVAGH.		24.2	29.5	96.1	Y.R..SL..ATVLCL		33.3	29.5	96.1	M.V.MN..VASIT..		40.0	29.5	96.1

Virus (accession number)	Id (%) Polyprotein (within Subgroup)	Class I: ATR #2668 (NS5)					Class IIb: ATR #3190 (NS5)					Class IIa: ATR #3286 (NS5)				
		IgM (e,a,b)	IgG (e,a,B)	Id (%) ATR	Id (%) Antig.	Id (%) within Subgroup	IgM (e,a,b)	IgG (E,a,b)	Id (%) ATR	Id (%) Antig.	Id (%) within Subgroup	IgM (E,a,b)	IgG (e,a,b)	Id (%) ATR	Id (%) Antig.	Id (%) within Subgroup
Zika (AAV34151, Africa)	100	SSPEVEETRTLRVLS		100	100		DRFAHALRFLNDMGK		100	100		HRRDLRLMANAICSAV		100	100	
Zika (AOS90225, USA)	96.4	.......A.......		93.3	95.7		....		100	95.7		..............S.		93.3	95.7	
Zika (ALU33341, Brazil)	96.4	.......A.......		93.3	95.6		....		100	95.6		..............S.		93.3	95.6	
Zika (AHZ13508, Fr. Polynesia)	96.5	.......A.......		93.3	95.8		....		100	95.8		..............S.		93.3	95.8	
Zika (AHL43504, Senegal)	99.0	....		100	98.9		....		100	98.9		....		100	98.9	
Dengue 1 (NP_059433)	55.5 (98.6)	PN.TI..G......K		60.0	65.8	98.7	....T..TA......		80.0	65.8	98.7	.......A........		93.3	65.8	98.7
Dengue 2 (NP_056776)	55.5 (97.7)	PN.T..AG......N		60.0	65.9	97.5	....S..TA......		80.0	65.9	97.5	.......A........		93.3	65.9	97.5
Dengue 3 (YP_001621843)	56.2 (98.7)	P..T...S..I...K		66.7	66.1	98.5	....N..LA......		80.0	66.1	98.5	.......AS.......		86.7	66.1	98.5
Dengue 4 (NP_073286)	56.3 (98.3)	.N.TI..G......K		66.7	67.7	98.5	E..GTS.L.......		66.7	67.7	98.5	.......ASM......		80.0	67.7	98.5
Yellow Fever (NP_041726)	46.6 (96.4)	..SVT.GE..V...D		53.3	60.6	96.5	...GL..SH..A.S.		60.0	60.6	96.5	.K..M..LSL.VS...		60.0	60.6	96.5
West-Nile (YP_001527877)	57.3 (98.6)	..A....H..I...E		73.3	69.9	99.6	....TS.H...A.S.		66.7	69.9	99.6	....		100	69.9	99.6
TBE (NP_043135)	41.5 (95.8)	PDAA..GE..RK.IL		33.3	56.8	98.5	...GK..Y.....A.		73.3	56.8	98.5	......TLGL..N...		73.3	56.8	98.5

Virus (accession number)	Id (%) Polyprotein (within Subgroup)	Class IIa: 3298 (NS5)				
		IgM (e,a,b)	IgG (e,A,b)	Id (%) ATR	Id (%) Antig.	Id (%) within Subgroup
Zika (AAV34151, Africa)	100	CSAVPVDWVPTGRTTWSIHGKGEW		100	100	
Zika (AOS90225, USA)	96.4	..S.....................		95.8	95.7	
Zika (ALU33341, Brazil)	96.4	..S.....................		95.8	95.6	
Zika (AHZ13508, Fr. Polynesia)	96.5	..S.....................		95.8	95.8	
Zika (AHL43504, Senegal)	99.0	....		100	98.9	
Dengue 1 (NP_059433)	55.5 (98.6)	...........S.......AHHQ.		79.2	65.8	98.7
Dengue 2 (NP_056776)	55.5 (97.7)	.....SH....S.......A.H..		79.2	65.9	97.5
Dengue 3 (YP_001621843)	56.2 (98.7)	......H....S.......AHHQ.		75.0	66.1	98.5
Dengue 4 (NP_073286)	56.3 (98.3)	.....TE.F..S.......AHHQ.		66.7	67.7	98.5
Yellow Fever (NP_041726)	46.6 (96.4)	S....TS...Q.............		83.3	60.6	96.5
West-Nile (YP_001527877)	57.3 (98.6)	......N............AG...		87.5	69.9	99.6
TBE (NP_043135)	41.5 (95.8)	N....A.............AS.A.		79.2	56.8	98.5

Table S3: FlaviMix-exclusive ATRs with high degree of residue identity between ZIKV and FlaviMix representatives (ATR class IV). FlaviMix-exclusive ATRs meeting the criterion of at least 80% residue identity with ZIKV are highlighted in grey. For other explanations see legend to table 1.

**Virus (accession number)**	Id (%) Polyprotein (within Subgroup)	ATR #718 (E)				ATR #1024 (NS1)				ATR #1618 (NS3)			
		Sequence	Id (%) ATR	Id (%) Antig.	Id (%) within Subgroup	Sequence	Id (%) ATR	Id (%) Antig.	Id (%) within Subgroup	Sequence	Id (%) ATR	Id (%) Antig.	Id (%) within Subgroup
Zika (AAV34151)	100	GSVGGVFNSLGKGIH	100	100	---	DGVEESDLIIPKSLA	100	100	---	DGDIGAVALDYPAGT	100	100	---
Dengue 1 (NP_059433)	55.5 (98.6)	..I....T.V..L..	73.3	58.9	98.5	N..L..EM....IYG	53.3	54.0	98.4	E.EV..I...FKP..	53.3	65.8	99.1
Dengue 2 (NP_056776)	55.5 (97.7)	..L....T.I..AL.	66.7	54.5	98.0	N..L..EM....N..	66.7	54.8	97.6	A.T....S..FSP..	60.0	66.4	98.3
Dengue 3 (YP_001621843)	56.2 (98.7)	......L.....MV.	80.0	58.7	98.5	N..L...M.......	80.0	55.7	98.7	T.E...I...FKP..	60.0	62.2	99.1
Dengue 4 (NP_073286)	56.3 (98.3)	.....L.T....AV.	73.3	56.7	98.3	N..L..QML....Y.	60.0	53.7	97.7	T.E....T..FKP..	60.0	67.0	98.7
Yellow Fever (NP_041726)	46.6 (96.4)	S.A..F.T.V.....	66.7	43.3	96.9	TS....EMFM.R.IG	40.0	47.6	96.7	G.E.........S..	80.0	51.8	97.9
West-Nile (YP_001527877)	57.3 (98.6)	.......T.V..AV.	73.3	54.0	98.9	..IL.......VT..	73.3	56.3	98.2	E.E....T..F.T..	66.7	67.7	98.7
TBE (NP_043135)	41.5 (95.8)	..A..FLS.I..AV.	53.3	39.1	98.8	AD.VD.E.FL.A...	46.7	43.5	98.5	GRKL..IPI.LVK..	33.3	45.6	97.0

**Virus (accession number)**	Id (%) Polyprotein (within Subgroup)	ATR #2836 (NS5)				ATR #3325 (NS5)			
		Sequence	Id (%) ATR	Id (%) Antig.	Id (%) within Subgroup	Sequence	Id (%) ATR	Id (%) Antig.	Id (%) within Subgroup
Zika (AAV34151, Africa)	100	SLVNGVVRLLSKPWD	100	100	---	EDMLMVWNRVWIEEN	100	100	---
Dengue 1 (NP_059433)	55.5 (98.6)	.M........T....	86.7	65.8	98.7	....S..........	93.3	65.8	98.7
Dengue 2 (NP_056776)	55.5 (97.7)	.M........T....	86.7	65.9	97.5	....T.......Q..	86.7	65.9	97.5
Dengue 3 (YP_001621843)	56.2 (98.7)	.MI....K..T....	73.3	66.1	98.5	....T..........	93.3	66.1	98.5
Dengue 4 (NP_073286)	56.3 (98.3)	.M.....K..T....	80.0	67.7	98.5	....K........D.	86.7	67.7	98.5
Yellow Fever (NP_041726)	46.6 (96.4)	.M....IKI.TY...	60.0	60.6	96.5	....E.......TN.	80.0	60.6	96.5
West-Nile (YP_001527877)	57.3 (98.6)	....	100	69.9	99.6	....E..........	93.3	69.9	99.6
TBE (NP_043135)	41.5 (95.8)	..I....K...W..N	73.3	56.8	98.5	....D.......LD.	80.0	56.8	98.5

Table S4: Comparing sets of experimentally identified ATRs (this study) and of those predicted on the basis by theoretical considerations [a,b]

ATR	Sequence	Region	Position		Epitope prediction	
			AAV3451	AMQ48981.1/ KF383119.1	Xu et. al, (AMQ48981.1) (a)	Homan et. al, (KF383119.1) (b)
**ATRs identified *via* ZIKV microarray chips, not predicted by Xu et. al., and Homan et. al.**						
25	SPFGGLKRLPAGLLL	C	25-39	25-39		
361	DSRCPTQGEAYLDKQSDTQYVCKRTLVDRG	E	361-390	361-390		
799	SKKETRCGTGVFVYN	NS1	799-813	803-817		
817	AWRDRYKYHPDSPRRLAAAVKQAW	NS1	817-840	821-844		
1024	DGVEESDLIIPKSLA	NS1	1024-1038	1028-1042		
1177	STSMAVLVAMILGGF	NS2	1177-1191	1181-1195		
1213	GGDVAHLALIAAFKVRPA	NS2	1213-1230	1217-1234		
1282	RAMAVPRTDNIALAILAALTPLAR	NS2	1282-1305	1286-1309		
1309	LVAWRAGLATCGGFMLLS	NS2	1309-1326	1313-1330		
1441	RLDVALDESGDFSLVEEDGPP	NS2	1441-1461	1445-1465		
1540	QEGVFHTMWHVTKGSALRSGEGRLDPY	NS3	1540-1566	1544-1570		
1585	AAWDGLSEVQLLAVPPGE	NS3	1585-1602	1589-1606		
1618	DGDIGAVALDYPAGT	NS3	1618-1632	1622-1636		
1654	IKNGSYVSAITQGKREEETPVECFEPSMLK	NS3	1654-1683	1658-1687		
1726	VAAEMEEALRGLPVR	NS3	1726-1740	1730-1744		
1741	YMTTAVNVTHSGTEI	NS3	1741-1755	1745-1759		
1780	YIMDEAHFTDPSSIA	NS3	1780-1794	1784-1798		
1840	ERAWSSGFDWVTDYSGKT	NS3	1840-1857	1844-1861		
1870	IAACLTKAGKRVIQL	NS3	1870-1884	1874-1888		
1888	TFETEFQKTK(N/H)QEWD	NS3	1888-1902	1892-1906		
1969	YLYGGGCAETDEDHA	NS4	1969-1983	1973-1987		
2029	TFVELMKRGDLPVWL	NS4	2029-2043	2033-2047		
2062	DGTTNNTIMEDSVPAEVWTKY	NS4	2062-2082	2066-2086		
2095	ARVCSDHAALKSFKE	NS4	2095-2109	2099-2113		
2170	IMLLGLLGTVSLGIF	NS4	2170-2184	2174-2188		
2203	LGASAWLMWLSEIEP	NS4	2203-2217	2207-2221		
2308	YAALTTFITPAVQHA	NS4	2308-2322	2312-2326		
2326	SYNNYSLMAMATQAGVLFGMGKGMPFYAWDFGV	NS4	2326-2358	2330-2362		
2401	QKRTAAGIMKNPVVDGIVVTDIDT	NS4	2401-2424	2405-2428		
2431	VEKKMGQVLLIAVAV	NS4	2431-2445	2435-2449		
2446	SSA(V/I)L(S/L)RTAWGWGEA	NS4	2446-2460	2450-2464		
2470	TLWEGSPNKYWNSSTATS	NS4	2470-2487	2474-2491		
2536	SALEFYSYKKSGITEVCREEARRALKDGVATGG	NS5	2536-2568	2540-2572		
2611	RKVQEVKGYTKGGPG	NS5	2611-2625	2615-2629		
2668	SSPEVEEARTLRVLS	NS5	2668-2682	2672-2686		
2791	MKIIGNRIERIRSEHAETWFFDENHPY	NS5	2791-2817	2795-2821		
2836	SLINGVVRLLSKPWD	NS5	2836-2850	2840-2854		
2866	YGQQRVFKEKVDTRV	NS5	2866-2880	2870-2884		
2926	LGAIFEEEKEWKTAV	NS5	2926-2940	2930-2944		
2956	REHHLRGECQSCVYN	NS5	2956-2970	2960-2974		
3037	EEMS(R/Q)(I/A)PGG(R/K)MYADDTAGWDTRISKFDL ENEAL	NS5	3037-3054	3041-3058		
3097	VLRPAEKGKTVMDII	NS5	3097-3111	3101-3115		
3169	GWDGLKRMAVSGDDCVVKPIDDRF	NS5	3169-3192	3173-3196		
3190	DRFAHALRFLNDMGK	NS5	3190-3204	3194-3208		
3205	VRKDTQEWKPSTGWD	NS5	3205-3219	3209-3223		

3229	HHFNKL(H/Y)LKDGRSIVVPC	NS5	3229-3246	3233-3250		
3286	HRRDLRLMANAICSS	NS5	3286-3300	3290-3304		
3298	CSAVPVDWVPTGRTTWSIHGKGEW	NS5	3298-3321	3302-3325		
3325	EDMLMVWNRVWIEEN	NS5	3325-3339	3329-3343		
3382	KNTVNMVRRIIG(D/E)EEKYMDYLSTQ	NS5	3382-3405	3386-3409		
**ATRs identified *via* ZIKV microarray chip and predicted by Xu et. al., and/or Homan et. al.**						
103	EKKRRGADTSVGIVG	C	103-117	100-114	100-110	100-107
130	RRGSAYYMYLDRNDAISF(A/P)TTLG(V/M)NKC(H/Y)(V/I)QIMDLGHMCD	prM	130-172	127-169	135-139/143-148	139-143
262	VAIAWLLGSSTSQKV	prM	262-276	262-276		269-273
304	GMSGGTWVDIVLEHGGCVTVMAQDKPTVDIELVTTTVSNMAEVRS	E	304-348	304-348	344-349	305-309
388	DRGWGNGCGLFGKGS	E	388-402	388-402	387-427	
433	VHGSQHSGMIVNDIGHETDENRAKVEITPNSPRAEAT	E	433-469	433-469	433-435/436-448	455-459/464-472
499	VHKEWFHDIPLPWHA	E	499-513	503-517	494-515	
526	EALVEFKDAHAKRQT	E	526-540	530-544	532-536	
592	SLCTAAFTFTKVPAETLHGTVTVEVQYAGTDGPCKV	E	592-627	596-631	608-616	
655	TENSKMMLELDPPFG	E	655-669	659-673	655-666/670-682	653-663
676	GVGDKKITHHWHRSG	E	676-690	680-694	670-682	
718	GSVGGVFNSLGKGIH	E	718-732	722-736	717-726	725-729
722	GGALNSLGKGIHQIF	E	722-735	726-739		725-729
742	LFGGMSWFSQILIGTLLMWLGLNTKNGSIS	E	742-771	746-775	746-752/756-759	
898	VNGLPHGWKAWGKSY	NS1	898-912	902-916	899-904/912-915	
931	KECPLKHRAWNSFLV	NS1	931-945	935-949	932-935/935-938	
967	LECDPAVIGTAVKGK	NS1	967-981	971-985	968-972	
1045	NTREGYRTQVKGPWHSEELEIRFEECPGTKVHV	NS1	1045-1077	1049-1081	1043-1051	
**ATRs only predicted by Xu et. al., and/or Homan et. al. but not found in this study**						
/	GGFRIVNMLKR	C	9-19	9-19	9-19	
/	SVGKK	C	71-75	71-75		71-75
/	NARK	C	96-99	96-99	96-99	
/	DEGVEPDDV	prM	179-187	179-187	179-187	
/	ARRSRRA	prM	210-216	210-216		210-216
/	SHSTRKL	prM	221-227	221-227		221-227
/	IGVSNRDFVEGMSGGTWVDVVL	prM	294-315	294-315	294-315	
/	QPENL	E	421-424	421-424		421-424
/	RIMLS	E	428-432	428-432	428-432	
/	SQHSGMIVNDTG	E	436-448	436-448	436-448	
/	ATLGG	E	464-468	468-472		468-472
/	AGADTG	E	513-518	517-522		517-522
/	GTPH	E	518-521	522-525	522-525	
/	VVLGSQEGAVHTALAGAL	E	542-559	545-562	545-562	
/	EMDGAKGRLS	E	562-570	565-573	565-573	571-579
/	HLKCRLKMDKL	E	574-584	577-587	577-587	
/	PCKVPAQMAV	E	624-633	628-637	628-637	
/	VGRLITANP	E	641-649	645-653	645-653	
/	HQIFG	E	732-736	736-740	736-740	
/	DVGC	NS1	791-794	795-798	795-798	
/	PVPVNE	NS1	895-900	899-904	899-904	
/	SYFVRAAKT	NS1	911-919	915-923	915-923	
/	DTLK	NS1	928-931	932-935	932-935	
/	LKVREDYSLE	NS1	959-968	963-972	963-972	

/	WYGMEIRP	NS1	1120-1127	1124-1131	1124-1131	
/	VLMTICGMN	NS2B	1470-1478	1474-1482	1474-1482	
/	PFAA	NS2B	1483-1486	1487-1490	1487-1490	
/	ALNTFTNLVVQLIRNMEAE	NS5	3126-3144	3130-3148	3130-3148	

a) **Xu X, Vaughan K, Weiskopf D, Grifoni A, Diamond MS, Sette A, Peters B.** 2016. Identifying Candidate Targets of Immune Responses in Zika Virus Based on Homology to Epitopes in Other Flavivirus Species. PLoS Curr **8**.

b) **Homan EJ, Malone RW, Darnell SJ, Bremel RD.** 2016. Antibody mediated epitope mimicry in the pathogenesis of Zika virus related disease. bioRxiv doi:doi.org/10.1101/044834.
